# Accelerating the transition from a linear to a circular healthcare sector: ESCH-R: study design and methodology

**DOI:** 10.3389/fpubh.2025.1542187

**Published:** 2025-03-26

**Authors:** Jilske Huijben, Erik van Raaij, Albert Wagelmans, Laura Piscicelli, Wei-Shan Shen, Bas van Vliet, Diederik Gommers, Dick Tibboel, Ellen Bakker, Florijn Dekkers, Redmer van Leeuwen, Jan Carel Diehl, Nicole Hunfeld

**Affiliations:** ^1^Department of Adult Intensive Care, Erasmus Medical Center, Rotterdam, Netherlands; ^2^Erasmus School of Health Policy and Management, Erasmus University Rotterdam, Rotterdam, Netherlands; ^3^Erasmus School of Economics, Erasmus University Rotterdam, Rotterdam, Netherlands; ^4^Copernicus Institute of Sustainable Development, Utrecht University, Utrecht, Netherlands; ^5^Environmental Technology Group, Wageningen University, Wageningen, Netherlands; ^6^Environmental Policy Group, Wageningen University, Wageningen, Netherlands; ^7^Rotterdam University of Applied Sciences, Research Centre Innovations in Care, Rotterdam, Netherlands; ^8^Research Office, University Medical Center Utrecht, Utrecht, Netherlands; ^9^Education Center, Faculty of Medicine, University Medical Center Utrecht, Utrecht, Netherlands; ^10^Department of Ophthalmology, University Medical Center Utrecht, Utrecht, Netherlands; ^11^Faculty of Industrial Design Engineering, Delft University of Technology, Delft, Netherlands; ^12^Department of Hospital Pharmacy, Erasmus Medical Center, Rotterdam, Netherlands

**Keywords:** sustainability, healthcare, circularity, hospital, planetary health

## Abstract

Due to the significant environmental impact of healthcare, there is an urgent need to accelerate its circular transition. We provide an overview of the ESCH-R project study design and methodology for accelerating the transition from a linear to a circular healthcare sector through the development and implementation of circular interventions in the Netherlands. Using a transdisciplinary approach, we will apply the 10-R ladder framework for a circular economy to hospitals. Methods are presented to analyze current clinical practices, policies and requirements for sustainable behavioral change, from material flows and operations to policy and regulations. We describe methods for the development of circular interventions, including business models, contract templates, and product redesigns. Finally, our approach to dissemination and education is presented. The described study design and methods can be used by other hospital (settings) to identify environmental hotspots for circular interventions in their own healthcare practice and for the cross-transfer of knowledge and anticipated challenges in implementing circular strategies. Ultimately, the ESCH-R project will deliver innovative, scalable approaches for hospitals to reduce procurement of raw materials, retain value of medical products, and reduce waste streams, CO_2_ emissions and pollution.

## Introduction

In the context of planetary health, the healthcare sector significantly contributes to worldwide environmental impacts, including air and water pollution, damage to ecosystems and biodiversity loss (1, 2). These in turn cause substantial health and societal risks in already overloaded healthcare systems, ranging from direct impacts due to extreme droughts, storms, or flooding—to indirect impacts like impaired mental health, malnutrition, and the further spread of infectious diseases (3). Therefore, the World Health Organization (WHO) has called for action to develop environmentally sustainable and climate resilient healthcare systems (4). Several global WHO initiatives, such as the Alliance for Transformative Action on Climate and Health (ATACH) and the Climate Change 26th Conference of the Parties (COP26) Health Programme place healthcare facilities at the forefront of health-promoting climate change communication and actions to achieve either low-carbon or net zero emissions health-care systems (5, 6). With the European Green Deal, the European Commission has set the goal for the European continent (including the healthcare sector) to become climate neutral and maximal circular by 2050 (7, 8). In the Netherlands, the healthcare sector is responsible for even around 13% of total materials extraction, 4–8% of total carbon emissions and 4% of direct and indirect waste generation (9–11). Therefore, there is an urgent need to accelerate its circular transition.

The majority of sustainability issues within hospitals can be attributed to the current design of the hospital supply chain as a linear system—with procurement and use of mainly single-use disposable consumables (12). Medical (single-use) consumables and their packaging require substantial amounts of energy and (raw) materials for their production, and their disposal leads to substantial waste generation. Current hospital waste streams consist of around 10–25% hazardous materials (e.g., infectious material, chemicals, or sharps) and 75–90% general hospital waste, of which 30–55% are plastics (13, 14). The share of single-use or even unused products (in their unopened, original packaging) is considerable, due to strict protocols focused on efficiency and infection prevention (13). A solution to reduce single-use consumables and waste streams would be to create a circular hospital system using the principles of the circular economy. A circular economy can be defined as “a waste-free economy that runs as much as possible on sustainable and renewable raw materials, and in which products and raw materials are reused” (12, 15, 16). Two fundamental strategies of resource cycling underlie the circular economy: the slowing resource loop (through reuse, the resource flow is slowed down, e.g., by the repair, refurbish or remanufacture of products to extend their product life) and the closing resource loop [through recycling, the resource loop is closed between post-use and production; (17)]. Besides these resource flows, other factors should also be considered in the hospital setting, such as maintaining optimal patient safety and outcomes, costs, healthcare quality, and working pressure on staff. Reducing the environmental impact in the healthcare sector is a growing field of interest, but successful design and implementation of circular interventions is difficult, as it requires systemic changes (at hospital and policy level) and adaptation of the whole hospital value chain (16, 18, 19). Therefore, novel transdisciplinary research is needed to take on this complex challenge from multiple perspectives.

The ESCH-R project (Evidence-based Strategies to create Circular Hospitals: Applying the 10-Rs framework to healthcare) is one of the first transdisciplinary projects on hospital sustainability in the world and consists of a unique consortium of stakeholders that represent the whole hospital value chain, including three university medical centers, six industry partners, eight universities, nine societal partners, and a national policy advisor. The goal of the ESCH-R project is to accelerate the adoption of circular interventions in hospitals, thereby lowering the ecological footprint of the healthcare sector in terms of CO_2_, material extraction and waste.

In this study, we will provide an overview of the ESCH-R project study design and methodology. First, we will describe the ESCH-R study design including the proposed framework, consortium partners and overall project management. Second, we will provide a detailed methodology of the various strategies to develop circular solutions.

## Methods

### Study design

#### Overview and 10-R framework

Within the ESCH-R project, we will develop strategies toward circular practices based on the following two aims: (1) to identify barriers and enablers toward circularity within the whole hospital ecosystem—from product development to product disposal, and across levels, from individual behavior to policy and regulation; (2) to co-design, implement and validate promising (evidence-based) interventions that are scalable to other hospitals—regionally, nationally and globally.

**Figure 1:**
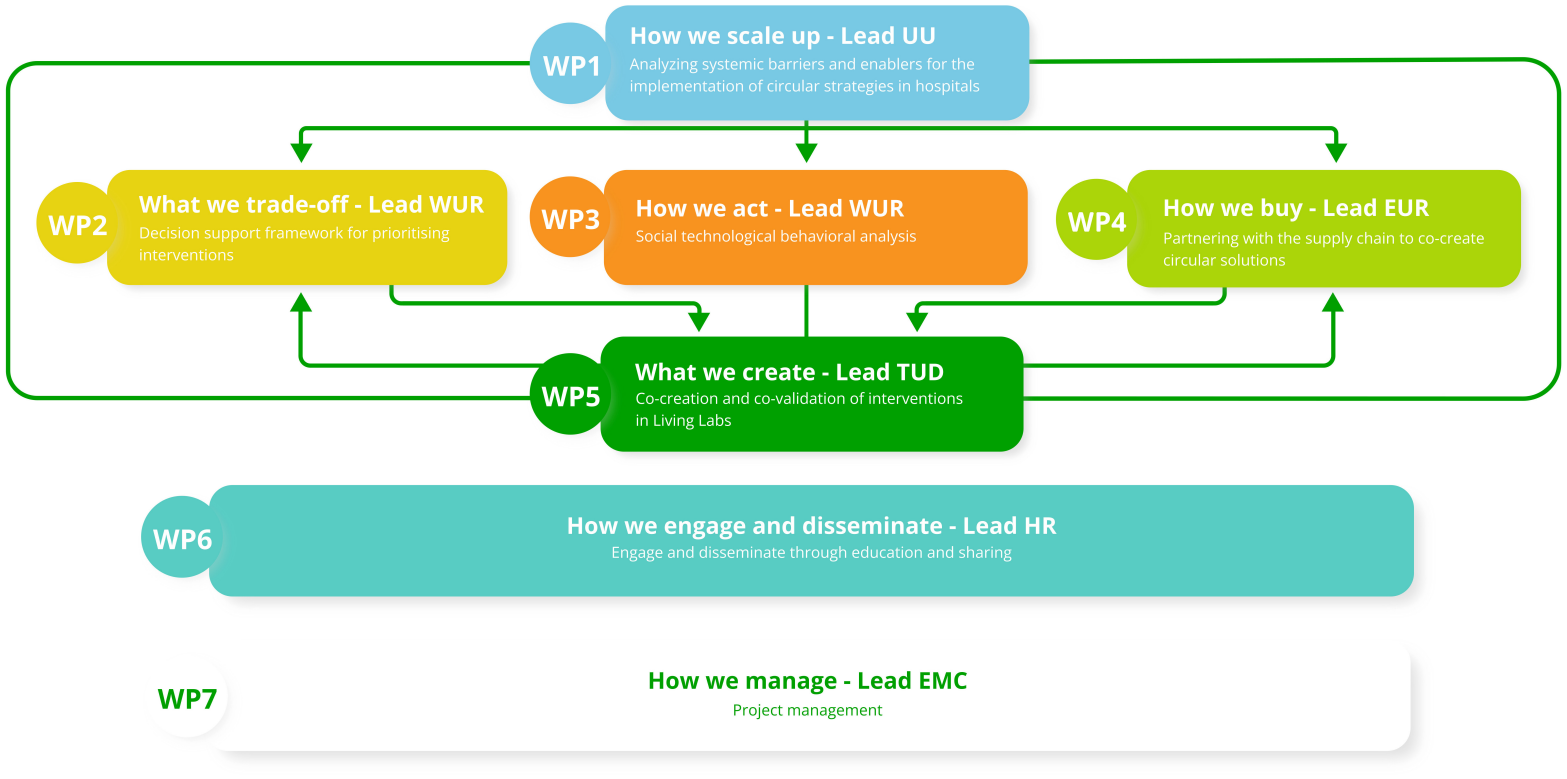
Overview of the ESCH-R work packages and connections. This figure shows the connections between the Work Packages (WPs) of the ESCH-R project. Both WP6 and 7 are overarching (ESCH-R project dissemination and management). EMC, Erasmus Medical Center; WUR, Wageningen University; EUR, Erasmus University; HR, University of Applied Sciences Rotterdam, TUD, Technical University Delft; UU, Utrecht University.

The ESCH-R project consists of seven Work Packages (WPs) to combine multiple perspectives and areas of expertise. The overarching WP1 aims to create an evidence-based roadmap for the successful implementation and upscaling of circular interventions in hospitals, integrating the knowledge gained in other WPs. WP2 aims to develop a decision support framework for prioritizing interventions. WP3 will analyze the hospital culture and practices of healthcare professionals on sustainable and circular behavior. WP4 will identify, design and validate business models and PSM (Purchasing and Supply Management) strategies that incentivize supply partners to develop circular solutions. WP5 entails the co-creation and co-validation of intervention cases with stakeholders in Living Labs. WP1–5 will address both the sector level challenges and the project level challenges of the intervention cases. WP6 will focus on the education of current and future healthcare staff, dissemination of results and engagement in best practices for a larger audience. Finally, WP7 covers overall project management (Figure 1).

**Figure 2:**
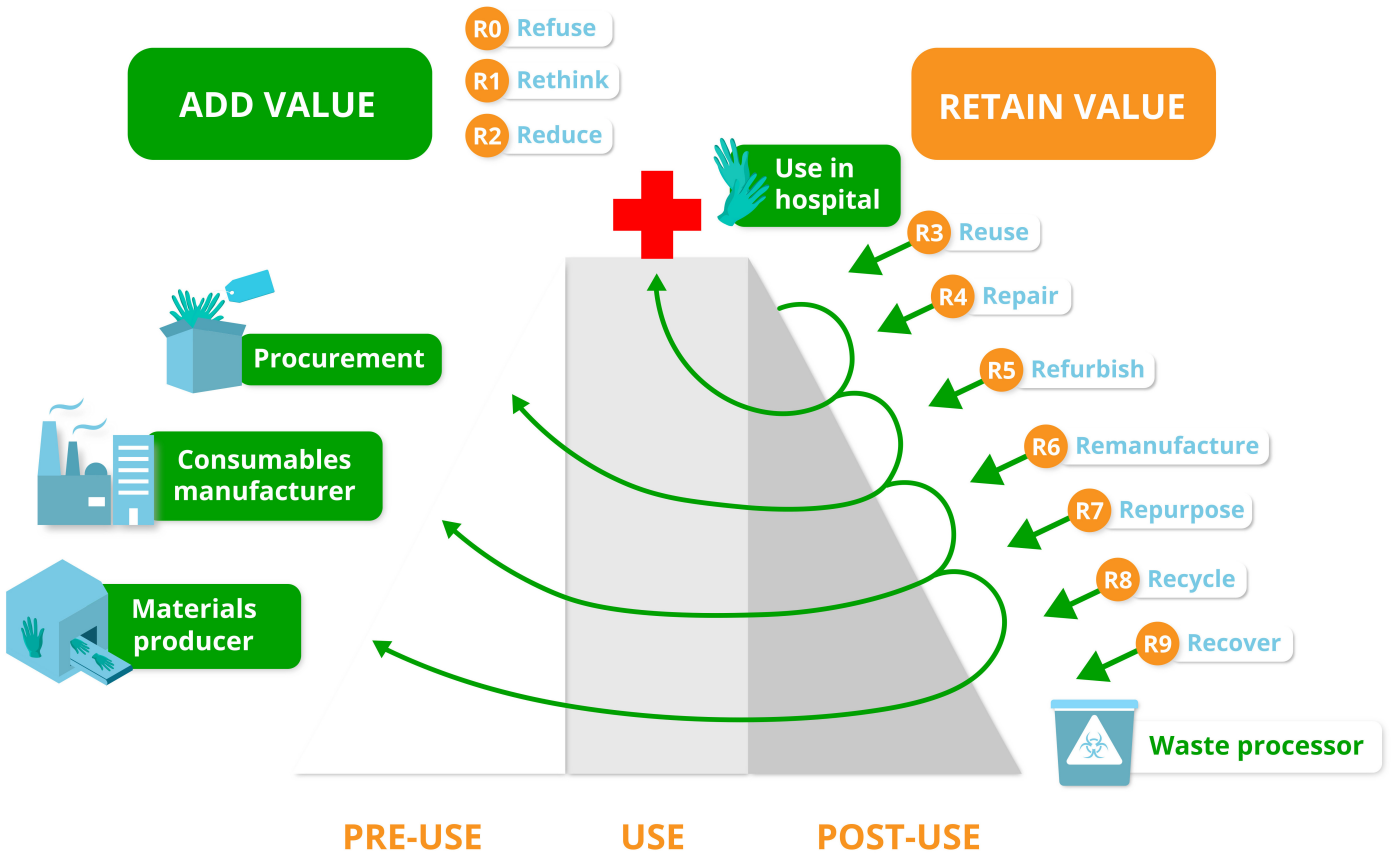
The 10R-value hill in the context of the ESCH-R project. This figure shows the various 10R Circular Economy strategies depicted along the value chain for medical consumables (left side of the figure). When a medical product falls further down the R value hill, the more value is lost (right side of the figure). The higher a medical consumable gets or stays on the hill the more value is added (green) or retained (orange). For each medical consumable in the context of the ESCH-R project the most optimal R strategy will be chosen and described to add or retain value.

The ESCH-R project will be structured by two frameworks in order to identify and cover all aspects of a circular economy. First, to identify sustainability issues and environmental hotspots, we will consider all seven pillars of the circular economy framework [materials, energy, water, biodiversity, human society and culture, health and wellbeing, and generating value; (20)]. Second, to support circular practices, several “R” ladder frameworks exist (12, 21, 22). In the context of the hospital, we will use the most extended version, the 10-R framework of circular strategies, as we can make use of all of the following resource value retention options: (0) Refuse, (1) Rethink, (2) Reduce, (3) Reuse, (4) Repair, (5) Refurbish, (6) Remanufacture, (7) Repurpose, (8) Recycle and (9) Recover (22). The 10-R strategies will be applied in various contexts within the ESCH-R project, such as the development of novel purchasing strategies, product redesign, and new business models. Also, behavioral interventions and process redesigns (including policy changes) are structured by the 10-R framework in the context of the hospital (Figure 2).

#### ESCH-R consortium

**Figure 3:**
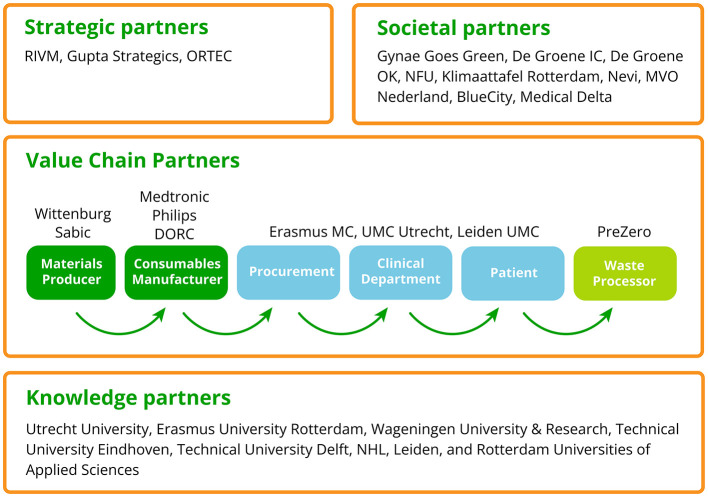
The ESCH-R consortium. This figure shows the strategic partners, societal partners, value chain partners and knowledge partners within the ESCH-R project, as the stakeholders that represent the whole hospital value chain. DORC, Dutch Opthalmic Research Center; Erasmus MC, Erasmus Medical Center; IC, Intensive Care; NFU, De Nederlandse Federatie van Universitair Medische Centra (Dutch Federation of University Medical Centers); UMC, Utrecht University Medical Center Utrecht; OK, Operatie Kamer (Surgery Room); RIVM, National Institute for Public Health and the Environment.

The ESCH-R consortium consists of a broad group of stakeholders to represent the whole hospital value chain; from materials producer and consumable manufacturer, through user, to waste processor, looking at safety, affordability, sustainability and product value. Overall, this requires strategic partners, societal partners, value chain partners and knowledge partners (Figure 3).

The strategic partners include a national policy advisor National Institute for Public Health and the Environment (RIVM), specialized in policies and regulations at a national level, a consultancy agency (Gupta strategists) specialized in healthcare and a data management company (ORTEC), specialized in data-driven decision making. The value chain partners include three academic medical centers (UMC Utrecht, Erasmus MC and Leiden UMC), who are medical consumables user and medical expert, and six industry partners (Philips, Medtronic, DORC, SABIC, Wittenburg and PreZero), who cover the chain from material supplier to waste processor (22). Knowledge partners are two social science universities (Utrecht University and Erasmus University Rotterdam), three technical universities (Wageningen University and Research, Eindhoven University of Technology, Delft University of Technology), which generate knowledge about circular design, business models, and environmental policy, and three universities of applied sciences (NHL Stenden, Leiden, and Rotterdam), which add technical expertise about medical consumables, Living Labs and education (knowledge dissemination) to the consortium. In addition, the ESCH-R project is supported by nine societal partners (Gynae Goes Green, De Groene IC, De Groene OK, NFU, Klimaattafel Rotterdam, Nevi, MVO Nederland, BlueCity and Medical Delta) who bring in a wide range of expertise and (public) networks, to increase societal impact.

#### Living labs

Two clinical Living Labs will be implemented for co-creation and co-validation of circularity interventions with representatives from all our stakeholders in two university hospitals (Erasmus MC and UMC Utrecht). A Living Lab can be defined as a user-centered, open innovation ecosystem based on a systematic user co-creation approach integrating research and innovation processes in real-life communities and settings (23). It is typically arranged with physical spaces in which stakeholders from academia, industry, public agencies and users collaborate on the creation, prototyping, validating and testing of new interventions, such as products, technologies, services and systems. In addition, a student led Living Lab will be installed at the Rotterdam University of Applied Sciences, to train a future generation of (healthcare) professionals in developing and implementing sustainable and circular practices in hospitals.

**Figure 4:**
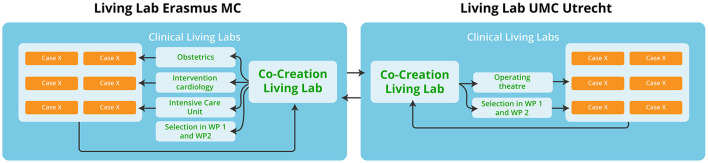
Identification and selection of environmental hotspots for co-creation in living labs. This figure shows the identification and selection of cases for the living labs within the two university medical centers. Based on the Material Flow Analyses (MFA) the environmental hotspots within the medical departments are identified (selection in WP1 and 2). The Operating Theater includes cases on robotic surgery and eye surgery. In total, we select six cases of high volume, low value medical consumables and six cases of low volume, high value medical consumables. Overall, we aim to design circular interventions (in co-creation workshops) that can be scalable to an (inter)national level.

We initiate one central co-creation Living Lab in each university hospital. The co-creation Living Lab is the venue where stakeholders can engage and co-create circular interventions. In the Living Labs data collection, experimenting and validation will take place. Clinical cases will be selected from medical departments that use medical consumables with high environmental impact and high consumption rates, such as the department of Obstetrics, Intensive Care Unit (ICU), Intervention Cardiology, and Operating Theater (including robotic surgery and eye surgery; Figure 4).

The clinical Living Labs will be combined with an overarching structure to engage ESCH-R project stakeholders, and we will organize multiple workshops with the ESCH-R consortium partners. Leiden UMC will function as a reference hospital to validate cases that have been pilot tested in Erasmus MC and UMC Utrecht.

#### Identification of circular intervention cases

Several strategies will be used to identify the most suitable intervention cases for circular solutions in the hospital. First, the cases in work package five will start with a literature review, since our approach is evidence-based. Based on literature we will proceed with the appropriate method; this can be a material flow analyses (MFA), (mini) Life Cycle Assessment (LCA), spend based CO_2_ analysis or care path analysis. The method will be selected based on transdisciplinary knowledge within our consortium.

The other work packages will also start with literature review and then describe circularity, behavior, circular procurement strategies and education practice, based on the topic of the work package.

#### Project and data management

The ESCH-R project management bodies are represented by the Project Board, Project Advisory Committee, and Work Package leaders. The Project Board oversees the project and is the decision-making body of the consortium. The Project Advisory Committee will monitor the progress of the project and includes representatives from the consortium and independent (legal and scientific) advisors. Work package leaders will guide the progress within their specific work packages.

Managerial implications concern mainly the transdisciplinary character of the project. With many different stakeholders involved from various backgrounds, we should engage them appropriately during the various phases of the project. This includes involvement of stakeholders in frequent mutual visits and co-creation workshops. Also, this requires frequent updates of the status of the project to inform and start collaborations.

The ESCH-R project started in January 2024 and has a project duration of 5 years. We expect the problem analysis (to identify barriers and enablers toward circularity within the whole hospital ecosystem) to be completed within the first 2 years. During the entire project, we will work on circular intervention strategies [to co-design, implement and validate promising (evidence-based) interventions]. Both qualitative data (e.g., interviews, focus groups, and cyclic design sessions) and quantitative data (e.g., surveys and product and goods consumption data) will be collected. Data will be stored within secure storage databases within institutes (for sensitive data) and in a dedicated Teams environment (for project documentation). Analytic software includes Atlas.ti, Covidence, SimaPro software, R and SPSS.

The ESCH-R project is funded by the Dutch Research Council NWO that funds research for all scientific disciplines in the Netherlands (NWA-ORC 2023, ref. NWA.1518.22.054). When intervention cases are tested in a real-time hospital setting, medical ethical approval will be obtained from the local medical ethical committees (METC).

### Methodology

#### Current practices, policies and circular behavior change

The academic literature on circular economy in the healthcare sector is still nascent, with significant gaps in comprehensive frameworks, interdisciplinary approaches and empirical data. Existing studies often rely on literature reviews (24), and limit their scope to (disposable or electronic) medical devices (12, 25–28). Adding to this body of knowledge, WP1 adopts the mission-oriented innovation systems (MIS) approach to analyze the current use of medical consumables in hospitals and investigate circular interventions within the Living Labs (29). The MIS framework enables a structured analysis of the ecosystem of actors and institutions that contribute to develop and diffuse innovative solutions aimed at addressing specific societal challenges, or “missions.” By mapping this network of stakeholders and their interactions, the MIS approach enables the identification of both systemic barriers and (policy) enablers that affect the adoption and diffusion of circular practices along the R-framework (22).

Literature reviews and interviews will be used to gather existing data on medical consumables and healthcare policies, and the institutional and policy settings within which healthcare practices are conducted. Next, we use interviews and participative observation methods to assess how healthcare professionals in ICU's and operation theaters handle materials in their professional practices (WP3). Using a Social Practice Theory framework, we will interview and observe healthcare professionals on their practices (their doings, the meanings attached to them and the materials involved), while performing those practices on the work floor (30).

After having gathered and analyzed data on systemic and practices level, WP1 will develop and implement circular interventions. Using a simulation model, informed by stakeholder input from the clinical Living Labs, we will identify key system factors and create a model to test the effects of potential circular interventions. This model will help assess how these interventions could be scaled up, providing insights where real-world testing is not feasible. A co-creation workshop with policymakers will refine the assessment, leading to an evidence-based roadmap and policy recommendations for the successful implementation and upscaling of circular practices in hospitals.

In parallel to workshops with policy makers, also co-creation sessions with healthcare professionals will be organized to design, test and evaluate behavioral interventions, their drivers and requirements in the use of materials in healthcare practices. Ultimately, the work of WP1 and WP3 aims to facilitate circular interventions for medical consumables on the work floor of participating hospital units and in broader hospital networks.

#### Hospital material flow characterization: from case-based to model-based

Designing the circularity transition in healthcare sector is complex and challenging due to (1) the complex and divers materials, products and equipment as well as the wide spectrum of possible circular interventions to each of them, (2) the aforementioned hospital-specific factors to be simultaneously considered, e.g., safety, cost, workload, etc. while designing and implementing circular transitions, (3) the interconnected stakeholders and stringent collective work flows that should be carefully changed and (4) the necessity to continuously maintain healthcare quality during the transition. Current literature addressing material flow analysis in healthcare sector employs mostly case studies and reports the status quo, which could not support a comprehensive evaluation and design of potential interventions (31–33). Simulation of future dynamics of these material flows with potential circular interventions in healthcare sector is important and demands a model-based approach. In this research, we envision developing a digital twin, i.e., a digital model replicating a physical process or system with continuously evolving data (34), to simulate the workflows of a hospital, from surgery level to entire hospital operation; the use of digital twin will enable the simulation and evaluation of the feasibility of each circular interventions and their consequences on all the hospital-specific factors and each workflow. Use of disposables and recyclable products and their potential alternatives will be monitored and recorded by the digital twin to simulate material, product and waste flows in hospitals, enabling identification of hotspots for interventions. A digital library of relevant circular interventions for hospitals, following the 10-R framework, will be built and categorized; life-cycle performances of these circular interventions will be incorporated based on existing literature or data from our industrial partners. Dynamic adaptive transition pathway analysis (35) for hospitals, building on the digital twin, circular intervention library and consideration of the other hospital-specific factors (input from other WPs), will be developed to enable the simulation of stepwise transition plan for hospitals to better guarantee the inclusion of all hospital-specific factors to be considered and to avoid the risk of discontinuing the healthcare service during the transition.

#### Circular and sustainable business models

Current business models in healthcare are mostly linear business models. Many medical devices and consumables are manufactured for single-use, which means that they often are incinerated or end up in landfill. Sometimes, multi-use variants are also available, but are not considered for a variety of reasons (28). Circularity implies that after use, medical devices and other products are cleaned and reused, are repurposed inside or outside of healthcare, are returned to the manufacturer for remanufacturing, or the materials that the products are composed of are recycled. Circularity can be achieved with current business models, but incentives for circularity may be much stronger if novel business models are developed. Business models, for instance, in which ownership is not transferred to the hospital and the reusability of products or their components becomes a stronger incentive for the manufacturer (25).

We will design, co-create and validate business models that create optimal incentives for circularity. We will use literature review to identify business models, interviews with a variety of stakeholders along the supply chain to identify strengths and weaknesses of current business models used in healthcare, co-create new business models with stakeholders, and validate new business models with buyers and suppliers.

#### Medical product redesign

Within the Living Labs we will design circular interventions with representatives from the whole hospital value chain, based on the environmental hotspots identified within WP1, 2 and 4 (Figure 4). This joint-innovation between stakeholders, including healthcare professionals, industry representatives, researchers and students should result in a blueprint which can be used for establishing a Circular Living Lab in other hospitals. Creative facilitation by means of co-creation workshops and design sprints will be applied to develop circular interventions. Co-creation and design can be seen as a culture where learning leads to innovation in a setting where the following principles are key: “Safety is everything,” “Fail forward to learn fast,” “Your parents don't work here,” “Talk to someone new,” and “All people have potential” (36).

We will use previously developed circular design approaches, including resource conservation design, slowing resource loops design, and whole systems design (37). In addition, we will develop a new Circular Design approach based on our experiences in the clinical living labs and ongoing reflection in clinical practice (mainly by healthcare workers). We will distinguish between low-value and high-value medical products. Low-value (high volume) medical single-use products, that cost 0–50 euro per product, include for example infusion bags, gloves, and syringes. We expect that the most promising circular strategies for low-value medical products are: Reduce, Rethink, Recycle and Recovery. This requires an organizational change in protocols and waste separation and less changes with regards to the business models. High-value (low volume) medical single use products cost 50–10,000 euro per product, such as robotic surgery disposables, catheters, and bronchoscopes. We assume the most promising circular strategies for high value medical products are Reuse, Rethink, Remanufacture, and Repair. Overall, we aim to achieve the reduction in CO_2_ emission by replacing single-use medical consumables with circular medical consumables and by using as few disposable medical consumables as possible (Refuse and Reduce).

The developed circular interventions will be co-validated in practice in two ways: validation in a healthcare setting within the Clinical Living Labs and with stakeholders along the value chain (i.e., material suppliers, medical consumables industry, and waste processors). This co-validation of the circular interventions can be in the early stage (ideation) with experimentation as well as in the final stage (finished design) with working prototypes. The final circular interventions will be assessed on their increased circularity (reduced environmental impact) in collaboration with WP2.

#### Multi-criteria decision-making

A variety of hospital processes, protocols and practices will need to be redesigned to enable circularity. These include day-to-day healthcare operations, material logistics in the hospital, waste handling, cleaning and sterilization procedures, and of course purchasing processes. Whenever a circular and more sustainable alternative process is considered, gains in sustainability need to be assessed against gains or losses in other performance areas. Similarly, if a more circular solution is considered in a purchasing process, it needs to be assessed vis-a-vis other solutions in terms of not just sustainability, but also other purchasing criteria (38).

In all these situations, multi-criteria decision-making (MCDM) is required. We start from a model in which sustainability is assessed vis-a-vis four hospital-specific factors: costs, health outcomes, safety (for patient and healthcare worker), and workload. Not all these dimensions can be quantified in all decision-making situations.

We will design, co-create and validate purchasing processes in which circularity and sustainability are considered amidst other purchasing criteria. We will start with a literature review on sustainable purchasing in healthcare. This is followed by interviews with purchasers and suppliers to identify the strengths and weaknesses of current purchasing procedures. After that, we will co-create purchasing strategies that create optimal incentives for suppliers to develop circular solutions.

Moreover, we will develop methods to model healthcare processes quantitatively and calculate trade-offs between environmental impact and performance on other hospital-specific factors. Such models will facilitate choices between alternatives and support decision-making in other work packages in this way. The novelty lies in the number of performance criteria to include in the models. In current studies, performance is typically considered on two or three hospital-specific factors, such as environmental impact and cost, or impact, cost and safety (infection risk).

#### Education for healthcare professionals

Despite all Dutch University Medical Centers have signed the Green Deal, sustainable and circular working of health care staff is not yet daily practice (39). Besides, in the majority of medical and nursing curricula sustainable and circular practices are not yet or not implemented (40–43). It is crucial that all healthcare staff and students should become aware of the importance of sustainable and circular practices through the dissemination and implementation of the results of the ESCH-R project in initial and advanced training courses.

Desk research will be conducted to collect preferably evidence informed education on sustainable and circular practices in hospital settings for healthcare staff. Nurses are the first in line of health education and in the ideal position to lead the way to increase awareness and address the health impacts of climate change. Therefore, there will be a special focus in the course material on nursing leadership in circular hospitals.

A network of medical and nursing educational institutions involved in the ESCHR project will be built. Participatory action and designed based research (44, 45) will be applied to conduct curriculum scans, develop or adapt existing course materials applicable to initial and advanced training courses. The guiding principle will be to find so-called links in initial and advanced training courses and curricula^8^ to which the ESCH-R project results can be integrated (46). We will co-create with user groups such as vocational, bachelor and master students, faculty staff and healthcare professionals. In this way education can be tailored, taking into account the increasing workload, and the shortage of healthcare staff (46, 47).

Dissemination of the evidence-based strategies as developed within the ESCH-R consortium will be performed both at national and international level throughout the project by organizing, developing and providing meetings, scientific webinars and conferences, podcasts and vlogs for the general public in collaboration with all work package leaders.

## Anticipated results

We expect to find that hospitals and the healthcare sector at large are severely “protocolized” due to prevailing health and safety regulations (based on infection prevention in particular). This means that we expect to encounter strict regulations for the (re-)use of materials in healthcare practices, affecting the implementation of certain changes toward circular use of materials that would work in other sectors, but not in healthcare. Yet, knowing the existing protocols and how they have come about over time may also generate insights into the changeability of protocols and hence the targeted healthcare practices.

However, sustainability transition in these protocols is only effective when health care workers show a behavioral change toward circularity, which we aim to support with newly developed behavior interventions. At management level, we expect to create a stepwise transition plan through the use of simulation of future dynamics. We strive to develop novel circular business models, e.g., where the supplier retains ownership of the product. We expect to create various sustainable design solutions targeted at both low-value and high-value medical products. Overall, circular behavior change and interventions can only be successfully implemented in the hospital setting, when maintaining costs, workload and health outcomes are ensured. The results of the ESCH-R project will be disseminated through various channels at national and international level and implemented in training courses and health-care curricula.

## Discussion

The ESCH-R project will provide novel circular approaches for hospitals to reduce the use and procurement of raw materials and virgin products, retain value of medical products and reduce waste streams and pollution. These approaches include new purchasing strategies, innovative business models, product redesign (medical consumables and their packaging), process redesign (including policy changes), and behavioral interventions. The ultimate goal would be to reduce the negative impact of hospitals on the environment—while maintaining current levels or improving on costs, patient safety, workload and health outcomes—and to improve global population health and planetary health. Results from the ESCH-R project should be scalable (inter)nationally and data should be standardized and harmonizable in the future.

Scalability of the results found in the ESCH-R project to other healthcare facilities can only be reached when circular strategies can be applied to material flow systems in various (international) hospital settings. Internationally, material flows and waste streams of medical consumables differ substantially between (high and low income) countries. For example, medical plastics represent 12% of healthcare waste in Peru, 26% in Kuwait and 46% in Italy (14). Still, global consumption of medical plastics is increasing, probably due to a growing demand in developing countries (13), as well as the use of virgin plastics due to the drop in oil prices (48). Substantial variation exists even between European countries regarding reprocessing (the top of the R-ladder) depending on whether reprocessing of single-use medical consumables is actually allowed by law. In addition, in practice, only a minority of medical devices—already approved to be reprocessed by the EU medical device (EU MDR) regulations—is currently reprocessed by health institutions (8). At the other end of the material flow, international waste stream processing options might differ, mainly with regard to recycling of plastics. To overcome these between-country differences, the ESCH-R project will map the individual material flows and redesign solutions of several widely used medical consumables. Thereby, (international) healthcare institutions can determine the best circular strategy within their country.

The ESCH-R project takes place in the Netherlands, where the current healthcare financial system is designed to reward care activities and interventions (and not reductions in care). Novel circular business models, based on services, could promote reprocessing and recycling options, thereby lowering healthcare costs and environmental impact. Still, several challenges exist in the healthcare sector to implement and adapt novel circular business models. First, the university hospitals in the Netherlands, as most hospitals in many other countries, are subject to public procurement law, which means that procurement is strongly regulated. This implies that tendering procedures need to be carefully prepared to obtain innovative sustainable solutions from the market. Second, the healthcare sector usually invests in a short time period (e.g., 2 years), while sustainable investments often take time to earn back. Finally, with a shift toward services instead of product contracts, follow-up of the investment is required; and the procurement team and end-users (healthcare professionals) should all be involved in both the early phase (e.g., regarding maintaining patient safety and health outcomes) and the implementation phase (e.g., regarding experienced barriers for use in clinical practice). Overall, both a system change and training is required before sustainability criteria in procurement of the hospital can be successfully applied.

In general, one of the challenges of sharing our methodology, is the standardization and future harmonization of data collection and methodology with other studies. Particularly, environmental assessments can be performed in different ways and are not standardized in healthcare. Another challenge of the ESCH-R project itself is to make circular solutions (tested in an academic hospital setting) applicable to other healthcare institutions, such as nursery homes, mental health care facilities, and outpatient clinics.

By describing circular redesign solutions of several widely used medical devices and sharing detailed methodology, we aim to achieve widespread application of our results.

To accelerate the transition toward a sustainable healthcare system, we first need to refuse, rethink and reduce our current treatment strategies (top strategies on the R ladder). Several treatment procedures that are currently performed routinely in clinical practice are not evidence-based and do not take the environmental impact into account. For example, at the ICU, some routine treatments are not evidence-based, while they have a significant impact on the environment and CO_2_ emissions. For instance, nebulization on demand appears to be clinically non-inferior to nebulization of acetylcysteine with salbutamol on routine (49). Furthermore, the sustainable healthcare transition will only be successful with the support of healthcare professionals and all stakeholders involved. This transition requires time for implementation of circular interventions in clinical practice and a culture change that should endure through the years. Therefore, we strive to involve current and future healthcare professionals from the early stage of the ESCH-R project. Within the living labs, current and future medical doctors, medical and technical students, and nurses are involved in both the evaluation and design stage of the clinical cases. Besides, the circular behavior change strategies will be used to develop evidence based educational strategies to be implemented in health care curricula and continuing education. Dissemination of all evidence-based strategies will be performed by all stakeholders both at a national and international level.

## Conclusion

The ESCH-R project is designed to accelerate the adoption of circular interventions in hospitals and thereby lower the ecological footprint of the healthcare sector in terms of CO_2_ emission, material extraction and waste. By involving stakeholders from multiple perspectives, the ESCH-R project has the potential to generate novel insights in circular solutions in all parts of the hospital supply chain. The novel circular solutions, like product redesign of medical consumables and their packaging, process redesign (including policy changes), and behavioral interventions should all consider improving on costs, patient safety, workload and health outcomes. By providing new purchasing strategies and innovative business models, hospital procurement is assumed to become more cost-effective, in addition to circular and sustainable. By involving current and future healthcare professionals in sustainable behavior change, the implementation of circular interventions should endure. To achieve international scalability of the results of the ESCH-R project, we will include circular redesigns solutions for several widely used medical consumables. Anticipated challenges for implementation of circular interventions are the variation in regulations on recycling or reprocessing possibilities between (country) health-care settings. The challenges for the implementation of novel business models are the current strongly regulated procurement, short-time investments and required transdisciplinary involvement during the whole procurement process. The results of the ESCH-R project will be disseminated through both scientific publications and articles, education, and webinars, podcasts, and articles for the general public.

## Data Availability

The original contributions presented in the study are included in the article/Supplementary material, further inquiries can be directed to the corresponding authors.
