# Supplementary material for: Accelerating the transition from a linear to a circular healthcare sector: ESCH-R: study design and methodology
Source: Front Public Health. 2025 Mar 26;13:1542187. doi: 10.3389/fpubh.2025.1542187 (PMC11978829; doi:10.3389/fpubh.2025.1542187)
Supplement: Supplementary file 1 [file Table_1.docx]

**ESCH-R consortium members**

Heidi Annala^1^, Willem Auping^2^, Conny Bakker^3^, Cathy van Beek^4^, Sabine Biesheuvel^5^, Hans de Brouwer^6^, Marlijn Caspers^7^, Myriam Cloodt^8,9^, Margot Cooijmans^10^, Marcel Crul^11^, Roeland Dijkema^12^, Anne van der Eijk^13^, Adriaan van Engelen^14^, Paula Goetz^2^, Pilar García-Gómez^15^ , Wilco van den Heuvel^15^, Saba Hinrichs-Krapels^2^, Arthur Haag^16^ , Niels Hagenaars^17^, Wouter Hehenkamp^18,19^, Iwan van der Horst^20,21^, Frank Willem Jansen^22^, Sanne Jansen^23^, Peter Joore^10^, Duygu Keskin^8^, Jasper Klasen^24^, Arjen van Klink^25^, Jotte de Koning^3^, Bertine Lahuis^26,27^, Mark Lazeroms^28^, Annemarie Leliveld^29^, Priscilla Lips^30^, Bas Maier^31^, Robert Metzke^32^, Ellen Moors^33^, Jacopo Parma^8^, Bas van Rijn^34^, Jelle Ruurda^35^, Vikrant Sihag^8^, Shalvi Thakur^15^, Gerrit Timmer^36^, Sacha Tensen^37^, Kim Verhaegh^38,39^, Susanne Waaijers - van der Loop^40^, Jeroen Wilschut^41^, Yuanyuan (Alice) Wu^42^, Yifan Yang^43^, Sophie van der Zee^15^, Joek van der Zwaan^33^

1. Environmental Policy Group, Wageningen University, The Netherlands
2. Faculty of Technology, Policy and Management, Delft University of Technology, Delft, The Netherlands
3. Faculty of Industrial Design Engineering, Department of Sustainable Design Engineering, Design for Sustainability, Delft University of Technology
4. Klimaattafel Rotterdam, Rotterdam, The Netherlands
5. BlueCity, Rotterdam, The Netherlands
6. SABIC, The Netherlands
7. NEVI, Zeist, The Netherlands
8. Department of Industrial Engineering and Innovation Sciences, Eindhoven University of Technology, Eindhoven, the Netherlands
9. Faculty of Management, Open University of the Netherlands, Heerlen, the Netherlands
10. Philips Foundation, Eindhoven, Netherland
11. NHL Stenden University of Applied Sciences, Leeuwarden, The Netherlands
12. DORC (Dutch Ophthalmic Research Center (International) B.V.), Zuidland, The Netherlands
13. Medical Delta, Delft, The Netherlands
14. Expertisecentrum Verduurzaming Zorg, Milieu Platform Zorgsector, Rotterdam, The Netherlands
15. Erasmus School of Economics, Erasmus University Rotterdam, Rotterdam, Netherlands
16. PreZero, Arnhem, The Netherlands
17. Gupta Strategists, Amsterdam, The Netherlands
18. Department of Obstetrics and Gynaecology, Amsterdam UMC, Amsterdam, the Netherlands
19. Centre for Sustainable Healthcare, Amsterdam UMC, Amsterdam, the Netherlands.
20. Department of Intensive Care Medicine, Maastricht University Medical Center, Maastricht, The Netherlands.
21. Department of Cardiovascular Research Institute Maastricht (CARIM), Maastricht, The Netherlands
22. Department of Obstetrics and gynaecology, Leiden University Medical Centre,
23. Department of Urology, University Medical Center Utrecht, The Netherlands
24. Department of Adult Intensive Care, Erasmus Medical Center, Rotterdam, the Netherlands.
25. Strategy, Research Centre of Business Innovation, Rotterdam University of Applied Science, Rotterdam, The Netherlands
26. Nederlandse Federatie van Universitair Medische Centra, Utrecht, The Netherlands
27. Human Resources, Radboud University Medical Center, Nijmegen, The Netherlands
28. Medtronic, Eindhoven, The Netherlands
29. Department of Urology, University Medical Center Groningen, Groningen, The Netherlands
30. Wittenburg, Zeewolde, The Netherlands
31. MVO Nederland, Utrecht, The Netherlands
32. Royal Philips Electronics, Amsterdam, Netherlands
33. Copernicus Institute of Sustainable Development, Utrecht University, Utrecht, the Netherlands
34. Department of Obstetrics and Gynecology, Erasmus MC University Medical Center, Rotterdam, The Netherlands
35. Department of Surgery, University Medical Center Utrecht, Utrecht, The Netherlands
36. ORTEC, Zoetermeer, The Netherlands
37. Human Resources and Healthcare, University Medical Centre Utrecht, Utrecht, The Netherlands
38. Leiden University of Applied Sciences, Department of Health, Leiden, the Netherlands
39. Alrijne Hospital, Department of Alrijne Academy, Leiderdorp, the Netherlands
40. Centre for Sustainability, Environment and Health, National Institute for Public Health and the Environment (RIVM), the Netherlands
41. Department of interventional Cardiology, Thoraxcenter, Cardiovascular Institute, Erasmus University Medical Center, Rotterdam, The Netherlands
42. Erasmus School of Health Policy & Management, Erasmus University Rotterdam, Rotterdam, The Netherlands
43. Environmental Technology Group, Wageningen University, The Netherlands
